# Maternal Circulating Exosomal miRNAs as Non-invasive Biomarkers for the Prediction of Fetal Ventricular Septal Defect

**DOI:** 10.3389/fgene.2021.717208

**Published:** 2021-09-09

**Authors:** Yuxia Jin, Ling Ai, Xiaojun Chai, Ping Tang, Weihua Zhang, Li Yang, Yue Hu, Ying Xu, Suping Li

**Affiliations:** ^1^Department of Prenatal Diagnostic, Jiaxing Maternity and Children Health Care Hospital, Jiaxing University, Jiaxing, China; ^2^College of Medicine, Jiaxing University, Jiaxing, China

**Keywords:** VSD, biomarker, serum exosomes, miRNA, prenatal diagnosis

## Abstract

**Objective:** This study aimed to identify maternal circulating exosomal miRNAs as potential non-invasive biomarkers for the early detection of fetal ventricular septal defects (VSDs).

**Methods:** In total, 182 pregnant women, comprising 91 VSD cases and 91 matched controls, were included in this study. Exosomes were isolated; dysregulated exosomal miRNAs were profiled using next-generation sequencing. Differential abundance of miRNAs was verified using quantitative real-time polymerase chain reaction (qRT-PCR). Diagnostic accuracy was evaluated by constructing receiver operating characteristic (ROC) curves.

**Results:** In total, 77 serum exosomal miRNAs were found to be differentially expressed in the VSD group compared to their expression in the control group. Among these, five downregulated exosomal miRNAs were validated using qRT-PCR. hsa-miR-146a-5p was identified to be capable of distinguishing VSD cases from controls (area under the ROC curve [AUC]: 0.997; *p* < 1.00E-05).

**Conclusion:** Circulating exosomal miRNAs, particularly hsa-miR-146a-5p, may be predictive biomarkers for the non-invasive prenatal diagnosis of fetal VSDs.

## Introduction

Congenital heart disease (CHD) is the most common type of birth defect and has an incidence of nearly 8 of every 1,000 live births worldwide (Erikssen et al., 2015). CHD represents approximately a third of all congenital anomalies and is the main cause of infant death (Hoffman and Kaplan, 2002). Ventricular septal defect (VSD), a major type of CHD, accounts for 20–30% of CHD cases. VSDs occur in nearly 1 of every 500 live births, and morbidity is even more common during the prenatal period (Bjornard et al., 2013). Consequently, early prenatal diagnosis is very important for timely surgical intervention and improved prognosis for both the mother and fetus.

Currently, the diagnosis of CHD is mainly dependent on fetal echocardiography during 22–28 weeks of pregnancy. However, CHD diagnosis with the use of echocardiography is partially limited by the skill of the operator, quality of equipment, and lack of process standardization. Therefore, there is an urgent need to identify early biomarkers in the maternal blood to enable accurate screening of fetal CHDs for more sensitive prenatal diagnosis. Recently, exosomal miRNAs have attracted considerable attention as disease biomarkers. In this study, we focused on identifying biomarkers of fetal VSDs by profiling maternal serum exosomal miRNAs.

Exosomes are extracellular vesicles (EVs) that contain macromolecules such as proteins, miRNAs, and mRNAs that can modulate target cell biology and function (Vlassov et al., 2012; Patil et al., 2019). Multiple lines of evidence indicate that exosomal miRNAs communicate between tissues and organs during organogenesis and repair (Hayashi and Hoffman, 2017; Hayashi et al., 2017). For example, exosomal miR-133a was found to improve cardiac function in a rat myocardial infarction model by reducing fibrosis and hypertrophy and by increasing vascularization and cardiomyocyte proliferation (Izarra et al., 2014).

Although exosomal miRNAs in maternal circulation have gained much interest for the detection of pregnancy-associated disorders, few studies have focused on the connection between exosomal miRNAs and fetal CHDs. We hypothesized that maternal serum exosomal miRNAs are potential candidate biomarkers for early prenatal detection of fetal VSDs.

In our study, we investigated miRNAs in EVs isolated from maternal serum as potential non-invasive screening biomarkers for fetal VSD. We analyzed exosomal miRNA profiles of maternal serum from pregnant women carrying fetuses with VSD as well as from pregnant women carrying normal fetuses as a control. The study identified hsa-miR-146a-5p as a potential novel early detection marker for VSD.

## Materials and Methods

### Study Design and Participants

The study was conducted on a retrospective cohort of pregnant women at 16–18 weeks of gestation who were admitted to the prenatal diagnosis center of Zhejiang Jiaxing Maternal and Child Health Care Hospital between January 2017 and January 2020. The cohort consisted of 91 pregnant women carrying fetuses with isolated VSD and 91 women carrying normal fetuses, as identified by prenatal cardiac ultrasound scanning. The disease and control groups were matched by maternal age, gestational age, and sampling date. All participants remained healthy and had no family genetic history. The clinical characteristics of the subjects are summarized in Table 1. The study was approved by the ethics committee of Jiaxing Maternal and Child Health Care Hospital, and informed consent was obtained from all participants.

**TABLE 1:** Clinical characteristics of pregnant women carrying fetuses with VSD and normal (control) fetuses in RNA sequencing and qRT-PCR analyses.

	RNA sequencing			qRT-PCR		
Characteristic	VSD (*n* = 36)	Control (*n* = 36)	*P*	VSD (*n* = 55)	Control (*n* = 55)	*p*
Gestational age (weeks)	16.74 ± 0.50	16.84 ± 0.46	0.38	16.75 ± 0.59	16.78 ± 0.59	0.76
Maternal age (years)	29.69 ± 4.66	28.61 ± 4.03	0.29	28.96 ± 4.79	28.61 ± 4.16	0.68

*VSD, ventricular septal defects.*

The study was divided into two stages. In the first stage (biomarker discovery), we created nine pools of serum samples from 36 VSD samples and 36 control samples, with each pool consisting of four samples. We then identified the differential miRNA profiles of the 2 groups by sequencing. In the second stage (biomarker validation), we conducted quantitative reverse transcription polymerase chain reaction (qRT-PCR) on samples from the remaining 55 pregnant women carrying fetuses with VSD and 55 women carrying normal fetuses to validate the miRNAs that were identified in the first stage. A receiver operating characteristic (ROC) curve was used to assess the accuracy of maternal serum miRNA in predicting fetal VSD.

### Serum Sample Preparation and Exosome Isolation

Each 4-mL serum sample pool was centrifuged twice (at 1,600 × *g* for 10 min and at 16,000 × *g* for 10 min) to remove cell debris and blood platelets. The supernatant was transferred to Eppendorf tubes for exosome isolation, miRNA extraction, and miRNA sequencing. Circulating exosomes were isolated from serum using Ribo^TM^ Exosome Isolation Reagent according to the manufacturer’s instructions (Guangzhou, China).

The remaining samples (55 VSD and 55 control) were used to validate the miRNA sequencing results via real-time qRT-PCR.

### Transmission Electron Microscopy (TEM)

Serum exosome samples (20 μL) were transferred to a copper mesh. Next, the sample was stained and fixed with 2% uranyl acetate for 1–10 min. The mesh was placed on a filter paper and air-dried. The morphological features of the exosomes were examined and visualized using TEM (Tecnai G2 Spirit Biotwin, FEI, United States) at a magnification of 44,000 × (200 nm).

### Nanoparticle Tracking Analysis (NTA)

The exosome particle quantity, size, distribution, and concentration were measured using NTA at VivaCell Biosciences with ZetaView PMX 110 (Meerbusch, Germany) and the corresponding software ZetaView 8.04.02.

Isolated exosome samples were appropriately diluted in 1 × phosphate-buffered saline (PBS), and NTA measurements were recorded and analyzed at 11 positions. The ZetaView system was calibrated using 110-nm polystyrene particles. The temperature was maintained at approximately 23–30°C.

### Western Blot Analysis

Exosomal protein was dissolved in radioimmunoprecipitation assay lysis buffer, and the total protein concentration was determined by a BCA protein assay. Approximately 60 μg of protein was subjected to 8–12% sodium dodecyl sulfate-polyacrylamide gel electrophoresis (SDS-PAGE) and transferred to a polyvinylidene fluoride membrane. The membrane was blocked for 1 h with 5% bovine serum albumin, and incubated with antibodies against CD63, CD9, and CD81(Abcam) at 4°C overnight, followed by incubation with a secondary antibody [goat anti-rabbit IgGH&L (HRP); Thermo Pierce] at 4°C for 1 h. Specific proteins were detected using SuperSignal^®^ West Dura Extended Duration Substrate according to the manufacturer’s instructions.

### Total RNA Extraction From Exosomes

Total RNA was extracted and purified from exosomes using the Liquid miRNA Kit/HiPure Serum/Plasma miRNA Kit (Megan, China). The quantity and integrity of exosomal RNA yield were assessed using Qubit^®^2.0 (Life Technologies, United States) and the Agilent 2200 TapeStation (Agilent Technologies, United States), respectively.

### MiRNA Library Construction and Sequencing

Exosomal RNA (50 ng) from each sample pool was used to prepare small RNA libraries using the NEBNext^®^ Multiplex Small RNA Library Prep Set for Illumina (NEB, United States) according to the manufacturer’s instructions. Small RNAs were reverse transcribed and amplified by PCR. Next, PCR products were sequenced by HiSeq 2500 (Illumina, United States) with a single-end 50-bp from Ribobio Co., Ltd. (Ribobio, China).

### Bioinformatics Analysis

To predict the target genes of the differentially expressed miRNAs, data from TargetScan, miRDB, miRTarBase, and miRWalk were integrated into our proprietary database. Only overlapping results of the four databases were accepted as potential target genes. Gene ontology (GO) analysis provides meaningful gene and gene products and covers three domains: biological processes (BP), cellular component (CC), and molecular function (MF). A *p*-value < 0.05 indicates a score representing significant GO-annotation enrichment for the differentially expressed genes. The Kyoto Encyclopedia of Genes and Genomes (KEGG) database was used to analyze signaling pathways related to target genes. The *p*-value was determined by Fisher’s exact test.

### Validation of Exosomal miRNA Expression by qRT-PCR

The Bulge-Loop^TM^ miRNA qRT-PCR Starter Kit (Ribobio, China) and miRNA-specific stem-loop primers were used to reverse transcribe total RNA. Based on the results of target gene prediction and enrichment analysis as well as literature, five exosomal miRNAs (hsa-miR-186-5p, hsa-miR-199a-3p, hsa-miR-146a-5p, hsa-miR-181a-5p, and hsa-miR-3158-3p) were selected to validate the miRNA sequencing results using an independent cohort of 110 exosomal samples (VSD, *n* = 55; control, *n* = 55) via qRT-PCR. Extracted RNA was reverse transcribed into cDNA, which was subjected to real-time qRT-PCR using a Bulge-Loop^TM^ miRNA qRT-PCR Starter Kit on a CFX Real-time PCR system (Bio-Rad, United States). Cel-miR-39 was added as an external control and normalized for technical variation between the samples, as described previously (Sohn et al., 2015). The relative expression of miRNAs was calculated using the 2^–△△*CT*^ method. Primer sequences of the five miRNAs are listed in Supplementary Table 1.

### Statistical Analysis

A two-tailed Student’s *t*-test was used to assess statistical significance; *p* < 0.05 and fold change > 2 were considered statistically significant. ROC curve and area under curve (AUC) were used to assess the sensitivity and specificity of the exosomal miRNAs for prenatal diagnosis of fetal VSD. All statistical calculations were performed using SPSS 23.0 and GraphPad Prism 6.0.

## Results

### Patient Characteristics

The total miRNA of the maternal serum exosomes from nine VSD sample pools and nine control sample pools was extracted for miRNA sequencing. The clinical characteristics of the 72 participants in the first stage and the other 110 participants in the second stage are shown in Table 1. There were no statistically significant differences between the two groups.

### Validation of Extracted Exosomes

**FIGURE 1:**
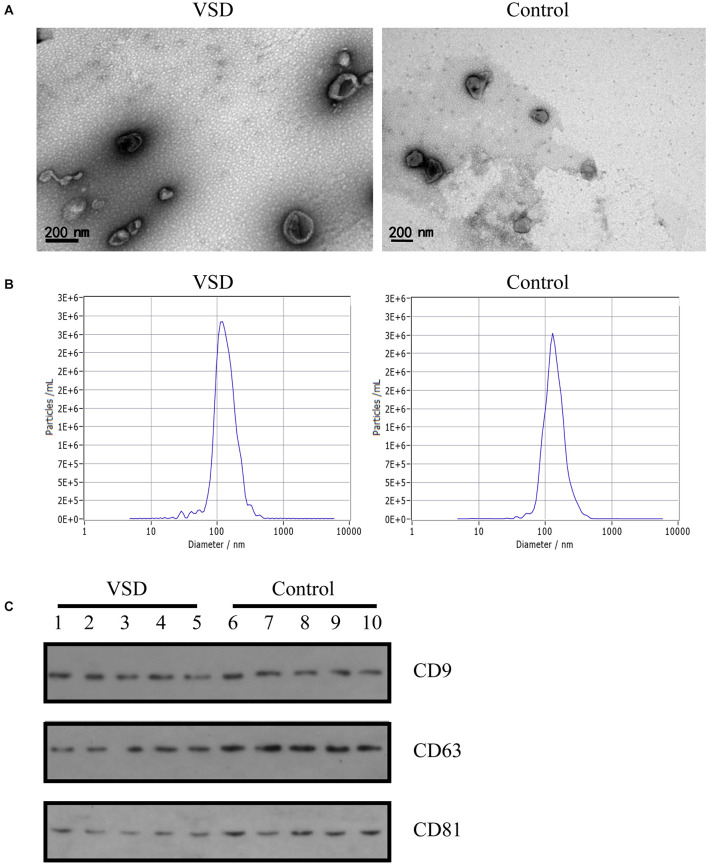
Identification of exosomes from maternal circulation. **(A)** Transmission electron microscopy results of exosomes from VSD and control samples. Scale bar: 200 nm. **(B)** Representative Nanoparticle Tracking Analysis (NTA) profiles for one VSD-extracellular vesicle (EV) and one Control-EV sample. **(C)** Exosomal membrane markers confirmed by western blotting (common markers enriched in exosomes: CD9, CD63, and CD81) in VSD and control groups (*n* = 5 per group).

Exosomes extracted from the nine VSD sample pools and nine control sample pools were assessed by TEM, NTA, and western blotting. As shown in Figure 1A, TEM analysis revealed a non-uniform distribution of cup-shaped structures in alveolar exosomes, with size ranging from 50 to 200 nm in diameter, in a dark background. NTA indicated that the isolated serum exosomes in the VSD samples had a mean diameter of 122.8 nm and those from the control samples had a mean diameter of 131.2 nm (Figure 1B); the differences between the two groups were not statistically significant. Furthermore, we selected serum exosomes (*n* = 5 per group) to evaluate the presence of the exosomal protein markers CD9, CD63, and CD81 through western blotting; the characteristics of the VSD and control samples were not significantly different (Figure 1C). Therefore, we can confirm that all obtained vesicles exhibited the major characteristics of exosomes and could be used in the following experiments.

### Differences in Circulating Exosomal miRNA Profiles Between Pregnancies With Fetal VSD and Those With Normal Fetuses

**FIGURE 2:**
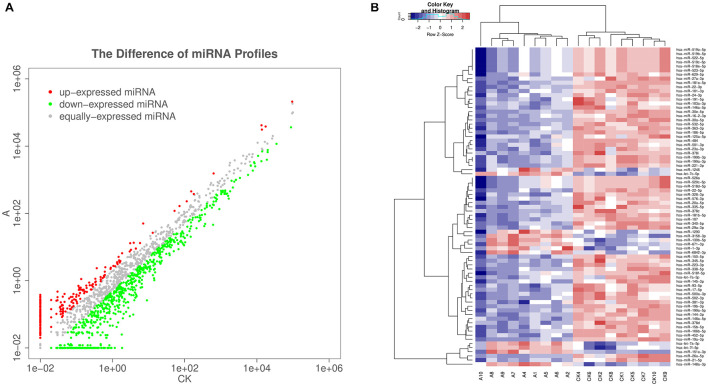
Sequencing-generated exosomal miRNA expression profiles for the serum of women carrying fetuses with VSD (*n* = 9) or normal fetuses (control, *n* = 9). **(A)** Scatter plots are used to illustrate the differential exosomal miRNA expression profiles between the VSD and control groups. Red, miRNAs with higher expression; green, miRNAs with lower expression; gray, miRNAs with equal expression. **(B)** Unsupervised hierarchical clustering analysis of miRNAs detected in all 18 samples (A, VSD group; CK, control group). Rows, miRNAs; columns, cases. The color legend across the top illustrates relative miRNA expression levels: blue for low expression and red for high expression (change > 2-fold as a cut-off; *p* < 0.05; expression level > 10).

The serum exosomal miRNA profiles of nine VSD sample pools and control sample pools were analyzed by sequencing. The differential exosomal miRNA expression profiles were analyzed between the VSD and control groups (Figure 2A). Unsupervised hierarchic clustering revealed a differential expression pattern between the two groups. In total, 77 miRNAs were identified to be differentially abundant between the VSD group and the control group, including 12 and 65 miRNAs that were upregulated and downregulated, respectively, in the VSD group (Figure 2B and Supplementary Table 2).

### GO Terms and KEGG Pathway Analysis of Differentially Expressed Exosomal miRNAs

**FIGURE 3:**
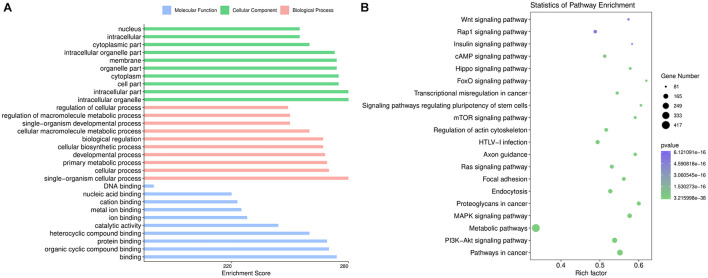
Bioinformatics analysis based on the sequencing results. **(A)** Gene Ontology enrichment results of cellular components, biological processes, and molecular functions. The enrichment score [–log10 (*p*-value)] of each term is shown on the horizontal axis and the term name on the vertical axis. **(B)** Kyoto Encyclopedia of Genes and Genomes enrichment results are displayed through a bubble chart. The horizontal axis shows the rich factor of each pathway. The number of genes involved in each pathway is indicated through the size of the bubbles while the color represents each *p*-value.

The top 10 GO categories for dysregulated miRNAs ranked by enrichment score [−log10(*p*-value)] are presented in Figure 3A. GO analysis indicated that the dysregulated miRNAs were mainly localized or interacted in the intracellular organelles, intracellular part, and cell part. These miRNAs are primarily involved in single-organism cellular processes, cellular processes, developmental processes, and single-organism developmental processes, but they also have roles in binding, organic cyclic compound binding, protein binding, catalytic activity, ion binding, and nucleic acid binding.

KEGG pathway analysis revealed 237 pathways that were significantly enriched (*p* < 0.05) for targets of deregulated miRNAs. As shown in Figure 3B, the target genes of the altered miRNAs are mostly involved in “Signaling transduction,” “Cellular community,” or other pathways. Our data showed that the top 20 pathways of altered miRNAs included the PI3K-Akt, MAPK, cAMP, and Hippo signaling pathways. These results are consistent with the fundamental functions of exosomes.

### Selection and Validation of Serum Exosomal miRNAs as Potential Circulating Biomarkers for Fetal VSD

**FIGURE 4:**
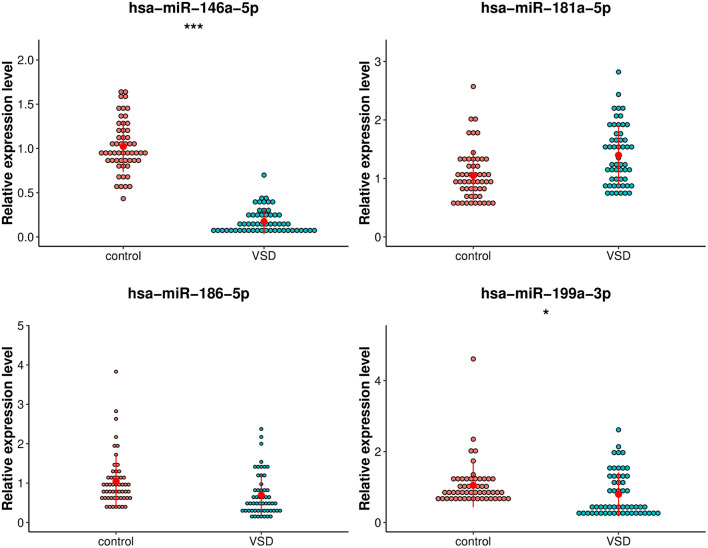
Comparison of the relative expression levels of exosomal hsa-miR-199a-3p, hsa-miR-146a-5p, hsa-miR-181a-5p, and hsa-miR-3158-3p between the VSD and control groups. Independent samples’ *t*-test indicated that exosomal hsa-miR-146a-5p was significantly downregulated in the VSD group than in the control group (*n* = 55 per group), ***p < 0.001; exosomal hsa-miR-199a-3p was significantly downregulated in the VSD group than in the control group (*n* = 55 per group), *p < 0.05.

The following criteria were applied to prioritize the candidate exosomal miRNAs: (1) reads > 100; (2) | log2FoldChange| > 2; (3) *p*-value < 1.00E-05. Fifteen miRNAs were selected, of which five exosomal miRNAs (hsa-miR-186-5p, hsa-miR-199a-3p, hsa-miR-146a-5p, hsa-miR-181a-5p, and hsa-miR-3158-3p) were shortlisted based on their fold changes and bioinformatics analysis as well as the literature associated with CHD (Kopke et al., 2015; Secco et al., 2018; Lei et al., 2019). The utility of these 5 miRNAs as biomarkers for fetal VSD was validated by qRT-PCR in an independent cohort of 110 serum samples (55 VSD samples and 55 control samples) (Table 1). However, hsa-miR-3158-3p was excluded from subsequent analyses because it showed CT values > 35 in qRT-PCR. As shown in Figure 4, of the remaining four miRNAs, hsa-miR-146a-5p and hsa-miR-199a-3p expression levels were significantly downregulated in the VSD group than in the control group, consistent with the sequencing results. The expression levels of the remaining two miRNAs (hsa-miR-186-5p and hsa-miR-181a-5p) were not significantly different between the two groups. Thus, we conducted ROC analysis for hsa-miR-146a-5p and hsa-miR-199a-3p.

**FIGURE 5:**
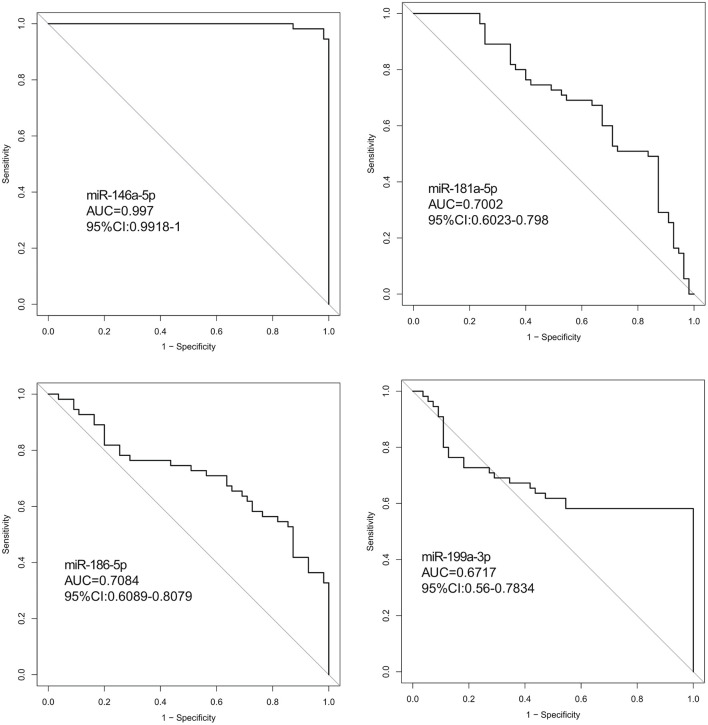
ROC curve analysis between the VSD and control groups. (ROC curve (AUC) analysis indicted that the expression values of serum exosomal miRNAs had good predictive accuracy). Exosomal hsa-miR-146a-5p in the cohort was the optimal cut-off point. ROC curve: Receiver operating characteristic curve.

Hsa-miR-146a-5p possessed the highest diagnostic power in discriminating fetuses with VSD and healthy fetuses, with an AUC of 0.997 (95%CI, 0.9918–1; cut-off < 0.49; sensitivity = 98.1%; specificity = 98.1%). The AUC of hsa-miR-199a-3p was 0.6717 (95%CI, 0.56–0.7834; cut-off < 0.55; sensitivity = 58.2%; specificity = 99.9%). These results indicate that hsa-miR-146a-5p can distinguish cases of fetal VSD from controls with high accuracy and may serve as an efficient and non-invasive biomarker for the prenatal detection of fetal VSD (Figure 5 and Table 2).

**TABLE 2:** Diagnostic efficiencies of serum exosomal miRNA in discriminating between fetuses with VSDs and controls.

miRNA	*n*	AUC	95%CI	*p*	Cut-off	Specificity	Sensitivity	Accuracy
miR-146a-5p	110	0.997	0.9918–1	<0.001	0.49086	0.9818182	0.9818182	0.9818182
miR-181a-5p	110	0.7002	0.6023–0.798	>0.05	1.46626	0.8727273	0.4909091	0.6818182
miR-186-5p	110	0.7084	0.6089–0.8079	>0.05	0.5602	0.8545455	0.5454545	0.7
miR-199a-3p	110	0.6717	0.56–0.7834	<0.05	0.55403	0.999996	0.5818182	0.7909091

*VSD, ventricular septal defects; AUC, area under the receiver operating characteristic curve.*

## Discussion

We retrospectively screened exosomal miRNAs from serum of pregnant women carrying fetuses with VSD or carrying normal fetuses at 16–18 weeks of gestation. In our study, we first analyzed the expression profile of exosomal miRNAs in maternal serum using sequencing technology, revealing 77 exosomal miRNAs that were differentially expressed in the serum of women carrying VSD fetuses compared to that in the serum of women carrying normal fetuses; of these 77 exosomal miRNAs, 12 were significantly upregulated and 65 downregulated in the VSD group. Following validation via qRT-PCR and ROC analysis, we demonstrated exosomal hsa-miR-146a-5p has high diagnostic performance in terms of sensitivity and specificity in differentiating cases of fetal VSDs from controls. These results imply that exosomal miRNAs may be useful predictive biomarkers for the non-invasive prenatal screening of fetal CHDs.

Previously, some studies have identified miRNAs in maternal circulation to be non-invasive biomarkers for prenatal diagnosis of CHDs (Zhu et al., 2013; Gu et al., 2019). However, currently, there is a tendency to use serum exosomal miRNAs as a promising non-invasive biomarker for detecting pregnancy-associated disorders (Salomon et al., 2017; Biro and Rigo Jr., 2018; Fallen et al., 2018; Biro et al., 2019). Compared with serum miRNAs, exosomal miRNAs are more sensitive, accurate, and specific. Research has indicated that miRNAs isolated from circulating serum exosomes are stable because the exosomal membrane provides a protective function against endogenous RNase (Whiteside, 2018). In addition, exosomal miRNAs are selectively wrapped by mediating proteins or proteins that identify miRNA motifs (Sanz-Rubio et al., 2018), explaining why exosomes contain different miRNAs in healthy and diseased individuals. Furthermore, exosomes are considered long-distance signal transporters that could play a significant role in communication between the embryo and mother (Hayashi and Hoffman, 2017). The specificity and stability of serum exosomal miRNAs as well as their functions provide unique opportunities for the advancement of non-invasive diagnostics, leading to the intense exploration of exosomal miRNAs in the maternal serum as molecular biomarkers for prenatal diagnosis of fetal CHD. To the best of our knowledge, the present study is the first to propose serum exosomal hsa-miR-146a-5p as a potential novel molecular biomarker for the detection of fetal VSD.

GO category annotation and KEGG pathway enrichment analyses were performed to gain insight into the biological functions and molecular mechanisms of deregulated exosomal miRNAs in the evolution of VSDs. The top 10 biological processes identified in the present study include developmental processes, particularly the single-organism developmental process. This result indicates that dysregulated miRNAs might participate in organism development during VSD pathogenesis. In KEGG pathway analysis, some classical pathways, such as the PI3K-Akt, MAPK, cAMP, mTOR, and Hippo signaling pathways (Assenza et al., 2018; Luo et al., 2018; Romano, 2019; Rowton et al., 2021), have been previously implicated in CHD, which indicates the reliability of our experimental data. These pathways play important roles in heart morphogenesis and all stages of cardiac development. In addition, we identified other pathways, such as signaling pathways regulating the pluripotency of stem cells or axon guidance, which provide the impetus for further study.

**FIGURE 6:**
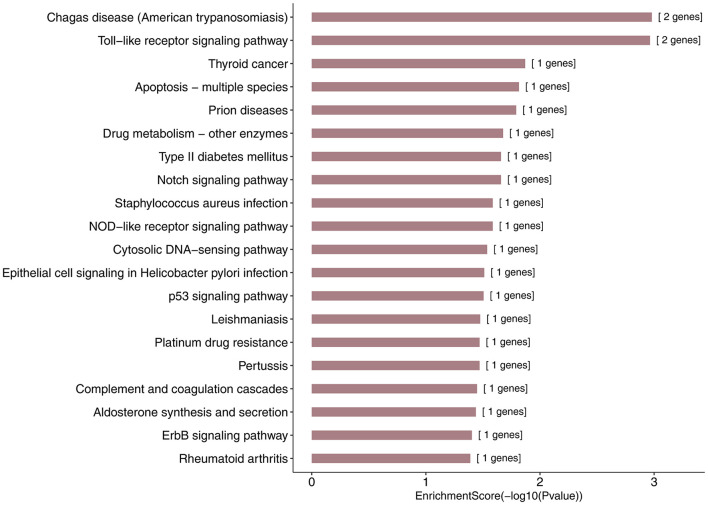
KEGG pathway analysis for the target genes of miR-146a-5p with the top 20 enrichment scores.

More specifically, we employed bioinformatics tools to predict KEGG pathways and potential target genes of hsa-miR-146a-5p. As indicated in Figure 6 and Table 3, TargetScan, miRDB, miRTarBase, and miRWalk predicted that *PMAIP1*, *NUMB*, *ERBB4*, *IRAK1*, and *CCL5* are potential target genes of hsa-miR-146a-5p; these genes are closely related to cardiac morphogenesis and development in five signaling pathways, including the Notch and ErbB signaling pathways. Our study predicts that hsa-miR-146a-5p participates in the Notch signaling pathway and that the putative target gene *NUMB* is involved in cardiac morphogenesis. In mice, *Numb* functions redundantly and has been shown to be essential for epicardial development, cardiac progenitor cell differentiation, outflow tract alignment, atrioventricular septum morphogenesis, myocardial trabeculation, and compaction (Wu and Li, 2015). Similarly, the ErbB signaling pathway plays an essential role in heart morphogenesis and in all developmental stages. ErbB4 plays an important role in cardiac development, regulates myocardial function, and participates in remodeling responses to physiology and pathology (Reichelt et al., 2017). In addition, we found that hsa-miR-146a-5p participates in various biological processes, such as inflammatory immune responses, and that its target genes *IRAK1* and *CCL5* are involved in the Toll-like receptor and NOD-like receptor signaling pathways. Nevertheless, future studies will focus on exploring the potential functions of hsa-miR-146a-5p and the underlying mechanisms to elucidate its association with CHDs.

**TABLE 3:** The top 20 most significant enriched KEGG pathway terms and the target genes of hsa-miR-146a-5p.

Term	ID	*p*	Gene IDs	Genes
Chagas disease (American trypanosomiasis)	hsa05142	0.00104666	3,654; 6,352	*IRAK1*; *CCL5*
Toll-like receptor signaling pathway	hsa04620	0.00108614	3,654; 6,352	*IRAK1*; *CCL5*
Pyrimidine metabolism	hsa00240	0.04690483	7,372	*UMPS*
Thyroid cancer	hsa05216	0.01349153	8,030	*CCDC6*
Apoptosis—multiple species	hsa04215	0.01527736	5,366	*PMAIP1*
Prion diseases	hsa05020	0.01616913	6,352	*CCL5*
Notch signaling pathway	hsa04330	0.02194705	8,650	*NUMB*
Drug metabolism—other enzymes	hsa00983	0.02106023	7,372	*UMPS*
Type II diabetes mellitus	hsa04930	0.02194705	5,581	*PRKCE*
Staphylococcus aureus infection	hsa05150	0.02592837	3,075	*CFH*
NOD-like receptor signaling pathway	hsa04621	0.02592837	6,352	*CCL5*
Cytosolic DNA-sensing pathway	hsa04623	0.02901436	6,352	*CCL5*
Epithelial cell signaling in Helicobacter pylori infection	hsa05120	0.03077363	6,352	*CCL5*
p53 signaling pathway	hsa04115	0.03121298	5,366	*PMAIP1*
Leishmaniasis	hsa05140	0.0334069	3,654	*IRAK1*
Pertussis	hsa05133	0.03384512	3,654	*IRAK1*
Platinum drug resistance	hsa01524	0.03384512	5,366	*PMAIP1*
Complement and coagulation cascades	hsa04610	0.03559613	3,075	*CFH*
Aldosterone synthesis and secretion	hsa04925	0.03647051	5,581	*PRKCE*
ErbB signaling pathway	hsa04012	0.03952494	2,066	*ERBB4*

The present study revealed that maternal serum exosomal hsa-miR-146a-5p was significantly downregulated in the VSD group compared to that in the control group, which indicates good diagnostic value for distinguishing cases of VSDs from normal cases. It has been reported that miR-146a-5p is highly enriched in human cardiac-resident mesenchymal progenitor cell (CPC) or cardiosphere-derived cell (CDC) exosomes (Barile et al., 2014). MiR-146a-5p has been shown to mediate some of the benefits of CDC exosomes in a mouse model of myocardial infarction or ischemia (Ibrahim et al., 2014). Well-known miR-146a-5p target genes include *Traf6*, *Smad4*, *Irak4*, and *Mpo*, all of which encode signaling mediators of the cell-death axes (Wang et al., 2013; Gao et al., 2015). Transfer of exosomal miR-146a-5p suppresses genes involved in cell death and may play a significant role in protecting against myocardial ischemia and inhibiting myocyte death (Milano et al., 2020). Thus, exosomal miR-146a-5p can be considered to mediate cardioprotective effects. Our results showed that exosomal miR-146a-5p in maternal serum was markedly decreased in the VSD group compared with that in the matched control group. We speculate that the potential protective effects of maternal serum exosomal miR-146a-5p on fetal heart deserve further detailed studies. We hypothesize that during embryogenesis, miR-146a-5p is transported across the placental barrier to the fetus via exosomes, where they are taken up by cardiomyocytes and contribute to the onset of cardiomyogenesis. This study is the first to confirm that the aberrant expression of maternal serum exosomal miR-146a-5p is associated with fetal VSD. Consistent with our data, miR-146a -5p was observed to be downregulated in myocardial infarction (Bukauskas et al., 2019), and recent studies have shown that a decrease in miR-146a-5p expression in the circulating plasma of pregnant women occurred prior to preeclampsia compared to women with normal pregnancies, and thus, miR-146a-5p could be used as a prognostic biomarker (Dayan et al., 2018). In this study, we further verified the diagnostic role of serum exosomal miR-146a-5p in the prenatal diagnosis of fetal VSD. However, the function and underlying mechanism of exosomal miR-146a-5p in fetal CHDs remain unclear. However, these findings on exosomal miR-146a-5p may provide a new direction for future studies and development of clinical strategies to prevent fetal CHD.

However, this study has some limitations. First, although our study successfully identified exosomal hsa-miR-146a-5p as a promising VSD-related biomarker for prenatal diagnosis, the number of pregnant women carrying fetuses with VSD enrolled in the present study was limited. Additionally, the heterogeneity and genetic background of pregnant women could impact the accuracy of the diagnostic value of exosomal miRNAs. Further studies are necessary to assess these exosomal miRNA signatures in a larger independent cohort of patients and controls. Second, we focused on fetal VSD in this study, and in future research, we will elucidate the prognostic value of exosomal miRNAs isolated from maternal serum in the detection of the diverse subtypes of CHD. Third, all dysregulated exosomal miRNAs were identified in the maternal blood, and thus, we could not determine the exact origins of the diagnostic serum exosomal miRNA biomarker. Whether they are derived from the embryos, placenta, or mother requires further investigation.

## Conclusion

This study demonstrated the diagnostic value of exosomal hsa-miR-146a-5p in the serum of pregnant women carrying fetuses with, with excellent sensitivity and specificity. Our findings highlight the potential clinical utility of serum exosomal miRNAs as an alternative for the diagnosis of fetal CHDs. Further large-scale validation and blinded clinical trials are required to confirm the potential applicability of these markers in prenatal VSD diagnosis, and additional exploration and optimization are imperative to uncover the underlying molecular mechanism of exosomal hsa-miR-146a-5p in VSD pathogenesis.

## Data Availability Statement

The original contributions presented in the study are publicly available in NCBI using accession number GSE176134.

## Ethics Statement

The studies involving human participants were reviewed and approved by the Ethics Committee of Women and Children’s Hospital Affiliated to Jiaxing University. The patients/participants provided their written informed consent to participate in this study.

## Author Contributions

YJ and SL conceived the study, participated to its design and coordination, and wrote the manuscript. LA and YX carried out the assays and participated to designing the study. XC, LY, and YH carried out laboratory tests. PT and WZ prepared the figures and tables. All authors read and approved the final manuscript.

## Conflict of Interest

The authors declare that the research was conducted in the absence of any commercial or financial relationships that could be construed as a potential conflict of interest.

## Publisher’s Note

All claims expressed in this article are solely those of the authors and do not necessarily represent those of their affiliated organizations, or those of the publisher, the editors and the reviewers. Any product that may be evaluated in this article, or claim that may be made by its manufacturer, is not guaranteed or endorsed by the publisher.

## References

[B1] Assenza M. R. Barbagallo F. Barrios F. Cornacchione M. Campolo F. Vivarelli E. (2018). Critical role of phosphodiesterase 2A in mouse congenital heart defects. *Cardiovasc. Res.* 114 830–845. 10.1093/cvr/cvy030 29409032

[B2] Barile L. Lionetti V. Cervio E. Matteucci M. Gherghiceanu M. Popescu L. M. (2014). Extracellular vesicles from human cardiac progenitor cells inhibit cardiomyocyte apoptosis and improve cardiac function after myocardial infarction. *Cardiovasc. Res.* 103 530–541. 10.1093/cvr/cvu167 25016614

[B3] Biro O. Rigo J. Jr. (2018). [The pathogenetic role and expression profile of microRNAs in preeclampsia]. *Orv. Hetil.* 159 547–556. 29611751 10.1556/650.2018.31025

[B4] Biro O. Fothi A. Alasztics B. Nagy B. Orban T. I. Rigo J. Jr. (2019). Circulating exosomal and argonaute-bound microRNAs in preeclampsia. *Gene* 692 138–144. 10.1016/j.gene.2019.01.012 30659946

[B5] Bjornard K. Riehle-Colarusso T. Gilboa S. M. Correa A. (2013). Patterns in the prevalence of congenital heart defects, metropolitan Atlanta, 1978 to 2005. *Birth Defects Res. A Clin. Mol. Teratol.* 97 87–94. 10.1002/bdra.23111 23404870

[B6] Bukauskas T. Mickus R. Cereskevicius D. Macas A. (2019). Value of serum miR-23a, miR-30d, and miR-146a biomarkers in ST-elevation myocardial infarction. *Med. Sci. Monit.* 25 3925–3932. 10.12659/msm.913743 31130720 PMC6556071

[B7] Dayan N. Schlosser K. Stewart D. J. Delles C. Kaur A. Pilote L. (2018). Circulating MicroRNAs implicate multiple atherogenic abnormalities in the long-term cardiovascular sequelae of preeclampsia. *Am. J. Hypertens.* 31 1093–1097. 10.1093/ajh/hpy069 29800045 PMC6132124

[B8] Erikssen G. Liestol K. Seem E. Birkeland S. Saatvedt K. J. Hoel T. N. (2015). Achievements in congenital heart defect surgery: a prospective, 40-year study of 7038 patients. *Circulation* 131 337–346. 10.1161/circulationaha.114.012033 25538230

[B9] Fallen S. Baxter D. Wu X. Kim T. K. Shynlova O. Lee M. Y. (2018). Extracellular vesicle RNAs reflect placenta dysfunction and are a biomarker source for preterm labour. *J. Cell. Mol. Med.* 22 2760–2773. 10.1111/jcmm.13570 29516617 PMC5908130

[B10] Gao M. Wang X. Zhang X. Ha T. Ma H. Liu L. (2015). Attenuation of cardiac dysfunction in polymicrobial sepsis by MicroRNA-146a is mediated via targeting of IRAK1 and TRAF6 expression. *J. Immunol.* 195 672–682. 10.4049/jimmunol.1403155 26048146 PMC4490963

[B11] Gu H. Chen L. Xue J. Huang T. Wei X. Liu D. (2019). Expression profile of maternal circulating microRNAs as non-invasive biomarkers for prenatal diagnosis of congenital heart defects. *Biomed. Pharmacother.* 109 823–830. 10.1016/j.biopha.2018.10.110 30551536

[B12] Hayashi T. Hoffman M. P. (2017). Exosomal microRNA communication between tissues during organogenesis. *RNA Biol.* 14 1683–1689. 10.1080/15476286.2017.1361098 28816640 PMC5731799

[B13] Hayashi T. Lombaert I. M. Hauser B. R. Patel V. N. Hoffman M. (2017). Exosomal MicroRNA transport from salivary mesenchyme regulates epithelial progenitor expansion during organogenesis. *Dev. Cell* 40 95–103. 10.1016/j.devcel.2016.12.001 28041903 PMC6720111

[B14] Hoffman J. I. Kaplan S. (2002). The incidence of congenital heart disease. *J. Am. Coll. Cardiol.* 39 1890–1900. 12084585 10.1016/s0735-1097(02)01886-7

[B15] Ibrahim A. G. Cheng K. Marban E. (2014). Exosomes as critical agents of cardiac regeneration triggered by cell therapy. *Stem Cell Rep.* 2 606–619. 10.1016/j.stemcr.2014.04.006 24936449 PMC4050492

[B16] Izarra A. Moscoso I. Levent E. Canon S. Cerrada I. Diez-Juan A. (2014). miR-133a enhances the protective capacity of cardiac progenitors cells after myocardial infarction. *Stem Cell Rep.* 3 1029–1042. 10.1016/j.stemcr.2014.10.010 25465869 PMC4264058

[B17] Kopke S. Buhrke T. Lampen A. (2015). miRNA expression in human intestinal Caco-2 cells is comparably regulated by cis- and trans-fatty acids. *Lipids* 50 227–239. 10.1007/s11745-015-3988-x 25612549

[B18] Lei Y. Guo P. Li X. Zhang Y. Du T. (2019). Identification of differentially expressed miRNAs and mRNAs in vestibular schwannoma by integrated analysis. *Biomed. Res. Int.* 2019:7267816. 10.1155/2019/7267816 PMC6594327 31309113

[B19] Luo G. P. Jian Z. Ma R. Y. Cao Z. Z. Zhu Y. Zhu Y. (2018). Melatonin alleviates hypoxia-induced cardiac apoptosis through PI3K/Akt pathway. *Int. J. Clin. Exp. Pathol.* 11 5840–5849. 31949670 PMC6963099

[B20] Milano G. Biemmi V. Lazzarini E. Balbi C. Ciullo A. Bolis S. (2020). Intravenous administration of cardiac progenitor cell-derived exosomes protects against doxorubicin/trastuzumab-induced cardiac toxicity. *Cardiovasc. Res.* 116 383–392. 31098627 10.1093/cvr/cvz108

[B21] Patil M. Henderson J. Luong H. Annamalai D. Sreejit G. Krishnamurthy P. (2019). The art of intercellular wireless communications: exosomes in heart disease and therapy. *Front. Cell Dev. Biol.* 7:315. 10.3389/fcell.2019.00315 31850349 PMC6902075

[B22] Reichelt M. E. O’Brien S. Thomas W. G. Headrick J. P. (2017). Transactivation of the epidermal growth factor receptor in responses to myocardial stress and cardioprotection. *Int. J. Biochem. Cell Biol.* 83 97–110. 10.1016/j.biocel.2016.12.014 28049018

[B23] Romano A. A. (2019). Growth and growth hormone treatment in noonan syndrome. *Pediatr. Endocrinol. Rev.* 16(Suppl. 2), 459–464. 31115197 10.17458/per.vol16.2019.r.growthhormonenoonan

[B24] Rowton M. Guzzetta A. Rydeen A. B. Moskowitz I. P. (2021). Control of cardiomyocyte differentiation timing by intercellular signaling pathways. *Semin. Cell. Dev. Biol.* 10.1016/j.semcdb.2021.06.002 34144893 PMC8968240

[B25] Salomon C. Guanzon D. Scholz-Romero K. Longo S. Correa P. Illanes S. E. (2017). Placental exosomes as early biomarker of preeclampsia: potential role of exosomal MicroRNAs across gestation. *J. Clin. Endocrinol. Metab.* 102 3182–3194. 10.1210/jc.2017-00672 28531338

[B26] Sanz-Rubio D. Martin-Burriel I. Gil A. Cubero P. Forner M. Khalyfa A. (2018). Stability of circulating exosomal miRNAs in healthy subjects. *Sci. Rep.* 8:10306. 10.1038/s41598-018-28748-5 PMC6037782 29985466

[B27] Secco I. Barile L. Torrini C. Zentilin L. Vassalli G. Giacca M. (2018). Notch pathway activation enhances cardiosphere in vitro expansion. *J. Cell. Mol. Med.* 22 5583–5595. 10.1111/jcmm.13832 30138533 PMC6201224

[B28] Sohn W. Kim J. Kang S. H. Yang S. R. Cho J. Y. Cho H. C. (2015). Serum exosomal microRNAs as novel biomarkers for hepatocellular carcinoma. *Exp. Mol. Med.* 47:e184. 10.1038/emm.2015.68 26380927 PMC4650928

[B29] Vlassov A. V. Magdaleno S. Setterquist R. Conrad R. (2012). Exosomes: current knowledge of their composition, biological functions, and diagnostic and therapeutic potentials. *Biochim. Biophys. Acta* 1820 940–948. 10.1016/j.bbagen.2012.03.017 22503788

[B30] Wang X. Ha T. Liu L. Zou J. Zhang X. Kalbfleisch J. (2013). Increased expression of microRNA-146a decreases myocardial ischaemia/reperfusion injury. *Cardiovasc. Res.* 97 432–442. 10.1093/cvr/cvs356 23208587 PMC3567787

[B31] Whiteside T. L. (2018). The emerging role of plasma exosomes in diagnosis, prognosis and therapies of patients with cancer. *Contemp. Oncol.* 22 38–40. 10.5114/wo.2018.73882 29628792 PMC5885072

[B32] Wu M. Li J. (2015). Numb family proteins: novel players in cardiac morphogenesis and cardiac progenitor cell differentiation. *Biomol. Concepts* 6 137–148. 10.1515/bmc-2015-0003 25883210 PMC4589147

[B33] Zhu S. Cao L. Zhu J. Kong L. Jin J. Qian L. (2013). Identification of maternal serum microRNAs as novel non-invasive biomarkers for prenatal detection of fetal congenital heart defects. *Clin. Chim. Acta* 424 66–72. 23707860 10.1016/j.cca.2013.05.010

