# Supplementary material for: Maternal Circulating Exosomal miRNAs as Non-invasive Biomarkers for the Prediction of Fetal Ventricular Septal Defect
Source: Front Genet. 2021 Sep 9;12:717208. doi: 10.3389/fgene.2021.717208 (PMC8458870; doi:10.3389/fgene.2021.717208)
Supplement: Supplementary file 1 [file Data_Sheet_1.docx]

Supplementary Material

# Supplementary Tables

Supplementary Table 1 Primer sequences for the five miRNAs

| miRNA | miRNA ID | miRNA_seq | F | R |
| --- | --- | --- | --- | --- |
| hsa-miR-146a-5p | MIMAT0000449 | UGAGAACUGAAUUCCAUGGGUU | F1-gcagtgagaactgaattcca | R1-ggtccagtttttttttttttttaacc |
|  |  |  | F2-gcagtgagaactgaattccat | R2-ggtccagtttttttttttttttaacc |
| hsa-miR-199a-3p | MIMAT0000232 | ACAGUAGUCUGCACAUUGGUUA | F1-cagacagtagtctgcacattg | R1-ggtccagtttttttttttttttaacc |
|  |  |  | F2-cagacagtagtctgcacattg | R2-caggtccagtttttttttttttttaac |
| hsa-miR-181a-5p | MIMAT0000256 | AACAUUCAACGCUGUCGGUGAGU | F1-cattcaacgctgtcggt | R1-ggtccagtttttttttttttttactca |
|  |  |  | F2-cattcaacgctgtcggt | R2-ggtccagtttttttttttttttactc |
| hsa-miR-186-5p | MIMAT0000456 | CAAAGAAUUCUCCUUUUGGGCU | F1-cgcagcaaagaattctcct | R1-ccagtttttttttttttttagcccaa |
|  |  |  | F2-cgcagcaaagaattctcct | R2-ggtccagtttttttttttttttagc |
| hsa-miR-3158-3p | MIMAT0015032 | AAGGGCUUCCUCUCUGCAGGAC | F1-cagaagggcttcctctct | R1-ggtccagtttttttttttttttgtc |
|  |  |  | F2-gcagaagggcttcctctc | R2-ggtccagtttttttttttttttgtc |

Supplementary Table 2 Significant changes in the expression of exosomal miRNAs in the serum of women carrying fetuses with VSD compared to those of women carrying normal control fetuses as determined by sequencing.

| miRNA | CK2 | A | log2(fold change) | up/down | p |
| --- | --- | --- | --- | --- | --- |
| hsa-miR-144-3p | 668.9689 | 39.3578 | -4.0872 | down | 5.96E-18 |
| hsa-miR-19b-3p | 398.1533 | 37.4078 | -3.4119 | down | 3.08E-18 |
| hsa-miR-19a-3p | 160.0367 | 17.9533 | -3.1561 | down | 1.83E-12 |
| hsa-miR-223-3p | 249.3767 | 33.7233 | -2.8865 | down | 4.86E-10 |
| hsa-miR-199a-5p | 285.3144 | 47.08 | -2.5994 | down | 1.34E-09 |
| hsa-miR-93-5p | 396.6433 | 68.5522 | -2.5326 | down | 1.31E-06 |
| hsa-miR-221-3p | 1307.286 | 226.4733 | -2.5292 | down | 3.96E-10 |
| hsa-miR-186-5p | 1684.219 | 291.7589 | -2.5292 | down | 4.73E-09 |
| hsa-miR-27a-3p | 8556.259 | 1527.653 | -2.4857 | down | 7.55E-06 |
| hsa-miR-181a-5p | 6093.791 | 1111.807 | -2.4544 | down | 1.51E-10 |
| hsa-miR-199a-3p | 1330.853 | 245.4456 | -2.4389 | down | 2.01E-09 |
| hsa-miR-199b-3p | 1330.853 | 245.4456 | -2.4389 | down | 2.02E-09 |
| hsa-miR-29a-3p | 613.7833 | 114.15 | -2.4268 | down | 5.91E-06 |
| hsa-miR-30e-5p | 1989.803 | 384.3967 | -2.372 | down | 2.95E-09 |
| hsa-miR-378c | 596.2289 | 116.1067 | -2.3604 | down | 0.00027316 |
| hsa-miR-338-5p | 163.7844 | 33.0667 | -2.3083 | down | 1.15E-07 |
| hsa-miR-148a-5p | 132.14 | 27.7889 | -2.2495 | down | 0.00649355 |
| hsa-miR-150-5p | 204.2011 | 43.6567 | -2.2257 | down | 4.53E-07 |
| hsa-miR-107 | 683.3444 | 147.1244 | -2.2156 | down | 3.64E-07 |
| hsa-miR-16-2-3p | 1642.344 | 354.6044 | -2.2115 | down | 5.76E-06 |
| hsa-miR-17-5p | 286.4689 | 63.0344 | -2.1842 | down | 0.00016424 |
| hsa-miR-146a-5p | 9604.698 | 2130.002 | -2.1729 | down | 3.33E-06 |
| hsa-miR-378i | 1127.336 | 251.58 | -2.1638 | down | 0.00699509 |
| hsa-miR-532-5p | 1500.966 | 338.9978 | -2.1465 | down | 8.16E-09 |
| hsa-miR-22-3p | 13652.53 | 3087.713 | -2.1446 | down | 1.29E-06 |
| hsa-miR-181b-5p | 791.1489 | 184.0667 | -2.1037 | down | 3.26E-07 |
| hsa-miR-363-3p | 1440.912 | 336.6556 | -2.0976 | down | 1.92E-07 |
| hsa-miR-145-3p | 297.3711 | 70.3311 | -2.08 | down | 8.45E-05 |
| hsa-miR-106b-5p | 86.9544 | 21.3089 | -2.0288 | down | 2.95E-06 |
| hsa-miR-502-3p | 255.8889 | 65.2044 | -1.9725 | down | 4.01E-05 |
| hsa-miR-378d | 114.7089 | 29.7244 | -1.9483 | down | 0.00987813 |
| hsa-miR-26a-5p | 38419.62 | 9988.708 | -1.9435 | down | 0.00507789 |
| hsa-miR-20a-5p | 399.07 | 106.0022 | -1.9125 | down | 0.00165678 |
| hsa-miR-22-5p | 422.2922 | 116.4911 | -1.858 | down | 0.00011421 |
| hsa-miR-328-3p | 584.8444 | 165.2411 | -1.8235 | down | 3.11E-05 |
| hsa-miR-484 | 797.8889 | 231.0522 | -1.788 | down | 9.39E-05 |
| hsa-miR-23a-3p | 844.04 | 245.1422 | -1.7837 | down | 0.00018263 |
| hsa-miR-381-3p | 248.9889 | 73.6944 | -1.7565 | down | 0.01955599 |
| hsa-let-7a-3p | 165.6044 | 49.0989 | -1.754 | down | 0.00862617 |
| hsa-miR-500a-3p | 274.5822 | 81.5178 | -1.7521 | down | 0.00115503 |
| hsa-miR-340-5p | 698.15 | 213.2811 | -1.7108 | down | 0.00857596 |
| hsa-miR-30a-5p | 1248.164 | 385.9389 | -1.6934 | down | 0.00877435 |
| hsa-miR-103a-3p | 7386.884 | 2311.46 | -1.6762 | down | 0.01161223 |
| hsa-miR-345-5p | 160.3989 | 50.57 | -1.6653 | down | 0.01337245 |
| hsa-miR-452-5p | 86.4378 | 27.9033 | -1.6312 | down | 0.00693842 |
| hsa-miR-21-5p | 21968.02 | 7113.677 | -1.6267 | down | 0.00872125 |
| hsa-miR-519b-5p | 2647.958 | 867.5389 | -1.6099 | down | 0.0007992 |
| hsa-miR-519a-5p | 2647.958 | 867.5389 | -1.6099 | down | 0.00079961 |
| hsa-miR-522-5p | 2647.958 | 867.5389 | -1.6099 | down | 0.00080327 |
| hsa-miR-519c-5p | 2647.958 | 867.5389 | -1.6099 | down | 0.00080353 |
| hsa-miR-518e-5p | 2647.958 | 867.5389 | -1.6099 | down | 0.0008052 |
| hsa-miR-523-5p | 2647.958 | 867.5389 | -1.6099 | down | 0.00080855 |
| hsa-miR-101-3p | 9495.679 | 3129.267 | -1.6014 | down | 0.00544935 |
| hsa-miR-191-5p | 9103.171 | 3037.373 | -1.5835 | down | 0.01580508 |
| hsa-miR-125a-5p | 1081.911 | 371.9478 | -1.5404 | down | 0.01250808 |
| hsa-miR-335-5p | 324.8633 | 112.7233 | -1.527 | down | 0.02618301 |
| hsa-miR-15b-5p | 99.2889 | 34.5767 | -1.5218 | down | 0.024968 |
| hsa-miR-501-3p | 705.5956 | 253.0456 | -1.4794 | down | 0.00529589 |
| hsa-miR-629-5p | 3034.377 | 1115.277 | -1.444 | down | 0.01081077 |
| hsa-miR-576-3p | 444.8833 | 171.0444 | -1.3791 | down | 0.01980787 |
| hsa-miR-24-3p | 11309.48 | 4374.338 | -1.3704 | down | 0.01732479 |
| hsa-miR-520c-5p | 458.8633 | 182.1122 | -1.3332 | down | 0.0304636 |
| hsa-miR-526a | 458.8633 | 182.1122 | -1.3332 | down | 0.0304904 |
| hsa-miR-518d-5p | 458.8633 | 182.1122 | -1.3332 | down | 0.03059792 |
| hsa-miR-518f-5p | 153.3822 | 61.1278 | -1.3272 | down | 0.0344107 |
| hsa-let-7a-5p | 19112.71 | 38492.54 | 1.01 | up | 2.09E-13 |
| hsa-let-7f-5p | 15149.91 | 30943.48 | 1.0303 | up | 1.60E-11 |
| hsa-miR-148a-3p | 104809.4 | 220936.5 | 1.0759 | up | 7.49E-13 |
| hsa-miR-6842-3p | 49.9522 | 108.0344 | 1.1129 | up | 2.76E-10 |
| hsa-miR-130b-5p | 112.6456 | 244.6822 | 1.1191 | up | 8.33E-11 |
| hsa-miR-1246 | 1849.691 | 4105.754 | 1.1504 | up | 0.00019318 |
| hsa-let-7c-5p | 671.1244 | 1553.193 | 1.2106 | up | 1.53E-11 |
| hsa-miR-1290 | 310.6333 | 741.71 | 1.2556 | up | 6.70E-06 |
| hsa-miR-671-3p | 73.3656 | 178.9833 | 1.2866 | up | 4.95E-13 |
| hsa-miR-151a-3p | 14495.96 | 43329.5 | 1.5797 | up | 6.86E-19 |
| hsa-miR-1-3p | 131.5411 | 405.2544 | 1.6233 | up | 9.64E-08 |
| hsa-miR-3158-3p | 133.1622 | 477.0611 | 1.841 | up | 4.02E-19 |
